# Genetic cluster analysis of SARS-CoV-2 and the identification of those responsible for the major outbreaks in various countries

**DOI:** 10.1080/22221751.2020.1773745

**Published:** 2020-06-11

**Authors:** Xuemei Yang, Ning Dong, Edward Wai-Chi Chan, Sheng Chen

**Affiliations:** a Department of Infectious Diseases and Public Health, Jockey Club College of Veterinary Medicine and Life Sciences, City University of Hong Kong, Kowloon, Hong Kong; b State Key Lab of Chemical Biology and Drug Discovery, Department of Applied Biology and Chemical Technology, The Hong Kong Polytechnic University, Kowloon, Hong Kong

**Keywords:** SARS-CoV-2, COVID-19, transmission, phylodynamic analysis, super-spreader

## Abstract

A newly emerged coronavirus, SARS-CoV-2, caused severe pneumonia outbreaks in China in December 2019 and has since spread to various countries around the world. To trace the evolution route and probe the transmission dynamics of this virus, we performed phylodynamic analysis of 247 high quality genomic sequences available in the GISAID platform as of 5 March 2020. Among them, four genetic clusters, defined as super-spreaders (SSs), could be identified and were found to be responsible for the major outbreaks that subsequently occurred in various countries. SS1 was widely disseminated in Asia and the US, and mainly responsible for outbreaks in the states of Washington and California as well as South Korea, whereas SS4 contributed to the pandemic in Europe. Using the signature mutations of each SS as markers, we further analysed 1539 genome sequences reported after 29 February 2020 and found that 90% of these genomes belonged to SSs, with SS4 being the most dominant. The relative degree of contribution of each SS to the pandemic in different continents was also depicted. Identification of these super-spreaders greatly facilitates development of new strategies to control the transmission of SARS-CoV-2.

## Introduction

A number of newly emerged coronaviruses such as the highly pathogenic severe acute respiratory syndrome coronavirus (SARS-CoV) and Middle East respiratory syndrome coronavirus (MERS-CoV) have caused serious respiratory and intestinal infections in human within the past two decades [1]. In December 2019, another new coronavirus, SARS-CoV-2, has emerged and caused outbreaks of lower respiratory tract infections, often with poor clinical outcome, in Wuhan, China. The virus, which has since spread to other cities in China and various countries worldwide [2], exhibited a high potential to undergo human-to-human transmission [3]. As of 7 May 2020, 3.7 million infections were recorded worldwide, among which a total of 3 million cases occurred in Northern America and Europe, and 83,000 cases were documented in China (https://www.gisaid.org/epiflu-applications/global-cases-betacov/). The WHO declared the risk of SARS-CoV-2 infection as “Very High” in late February (https://www.who.int/docs/default-source/coronaviruse/situation-reports/20200228-sitrep-39-covid-19.pdf).

The genomic characteristics of SARS-CoV-2 have been elucidated by means of phylogenetic, structural and mutational analyses by scientists around the world [4]. High-throughput sequencing showed that SARS-CoV-2 was a novel betacoronavirus which resembled SARS-CoV at around 79.5% sequence identity [5,6]. A recent study indicated that SARS-CoV-2 was 96% identical to a bat coronavirus RaTG13 (accession: MN996532) at the genomic level, suggesting that bat might be a natural host of SARS-CoV-2 [7]. GISAID is a platform for sharing genetic data of influenza. Currently, a rapidly increasing number of SARS-CoV-2 genomic sequences are being deposited into this database from laboratories around the world [8]. On the other hand, some recent studies also suspected that Malayan pangolins (*Manis javanica*) could be the intermediate host of this new coronavirus, since the amino acid sequence of the S protein of coronaviruses derived from Malayan pangolins illegally imported to Guangdong Province of China, as well as coronaviruses harboured by pangolins in Guangxi province of China, exhibited very high homology with the S protein sequence of SARS-CoV-2, even though the overall homology between SARS-CoV-2 and RaTG13 is still the highest [9]. However, due to the inability to detect or isolate SARS-CoV-2 from pangolins in the Wuhan Huanan seafood wholesale market, the site in which the first batch of infected patients had commonly visited, the theory of pangolins being the culprit of the Wuhan pneumonia outbreak is not substantiated. The intermediate host for SARS-CoV-2 therefore remains a mystery. In fact, it remains unclear if the Huanan Seafood Wholesale Market is the origin of this outbreak as some of the earliest cases were confirmed to have no linkage with this market. It is necessary to identify the source(s) of virus(es) that caused this outbreak to design more effective control measures to stop the continuous worldwide transmission of these highly contagious viruses. With more sequences being released, we can obtain a more comprehensive view on the genomic features of this virus through in-depth sequence analysis. One recent study has analysed over 100 available genome sequences and showed that sequences belonging to different genetic clusters have evolved [10]. In this work, we retrieved and analysed the publicly shared genome sequences of SARS-CoV-2 available as of 26 March 2020 to investigate the genetic diversity and phylodynamics of these viruses. We identified four distinct viral clusters which apparently exhibit high mutation rate and have become the most dominant viruses that prevailed in the global pandemic that started in January, 2020. Results in this study should provide valuable insight into key factors that scientists and clinicians need to consider in the control of the SARS-CoV-2 pandemic, in particular the transmission fitness and differential virulence levels of different viral strains.

## Materials and methods

### Sequence analysis, alignment and mutation identification

A total of 343 full-length SARS-CoV-2 genomes available in the GISAID platform (https://platform.gisaid.org/) as of 10 March 2020 with 5 March 2020 as cut-off date were downloaded [8]. A total of 247 sequences with high sequence quality as noted in the GISAID database were included for further analysis after removing sequences containing little temporal signal and thus are not unsuitable for inference using phylogenetic molecular clock models. Information regarding the date and country of isolation were also retrieved from the GISAID platform. The annotated reference genome sequence of the SARS-CoV-2 isolate Wuhan-Hu-1 (accession: NC_045512.2) was downloaded from the NCBI GenBank database. All genomes were annotated by GATU Genome Annotator [11] using the SARS-CoV-2 isolate Wuhan-Hu-1 (NC_045512.2) as reference [12]. Nucleotide and amino acid mutations of all genome and separate proteins were analysed by blast (https://blast.ncbi.nlm.nih.gov/) using the sequence of strain Wuhan-Hu-1 as reference.

### Phylodynamic analysis

Global genomic surveillance of SARS-CoV-2 was implemented by means of the automated phylogenetic analysis pipeline Nextstrain, which generates an interactive visualization integrating a phylogeny with sample metadata such as geographic location or isolation date [13]. The pipeline involved the sequence alignment module with MAFFT [14], phylogenetic analysis with IQ-TREE [15], maximum-likelihood phylodynamic analysis with Treetime [16], identification of nucleotide and amino acid mutations with Augur, and result visualization with Auspice [13]. The outputs were edited by Inkscape 0.91 [17].

### Phylogenetic analysis

Alignment of the complete genome sequences of SARS-CoV-2 strains was conducted with MAFFT v7.310 [14]. Phylogenetic tree of all SARS-CoV-2 strains was built with RAxML version 8.2.4 [18]. The tree was edited by iTOL [19].

### Quick identification of the types of SARS-CoV-2 genomes in the database

All complete genomes available as of 28 March 2020 with 26 March 2020 as cut-off date in the GISAID database were downloaded. Single Nucleotide Polymorphisms (SNPs) calling were performed by Snippy (https://github.com/tseemann/snippy) using Wuhan-Hu-1 as reference. Super-spreader clusters were classified according relative variants. A total of 1539 qualified genomes submitted after 29 February 2020 were included.

## Results

### Phylodynamics analysis of genome sequences of SARS-CoV-2 strains collected worldwide

**Figure 1.**
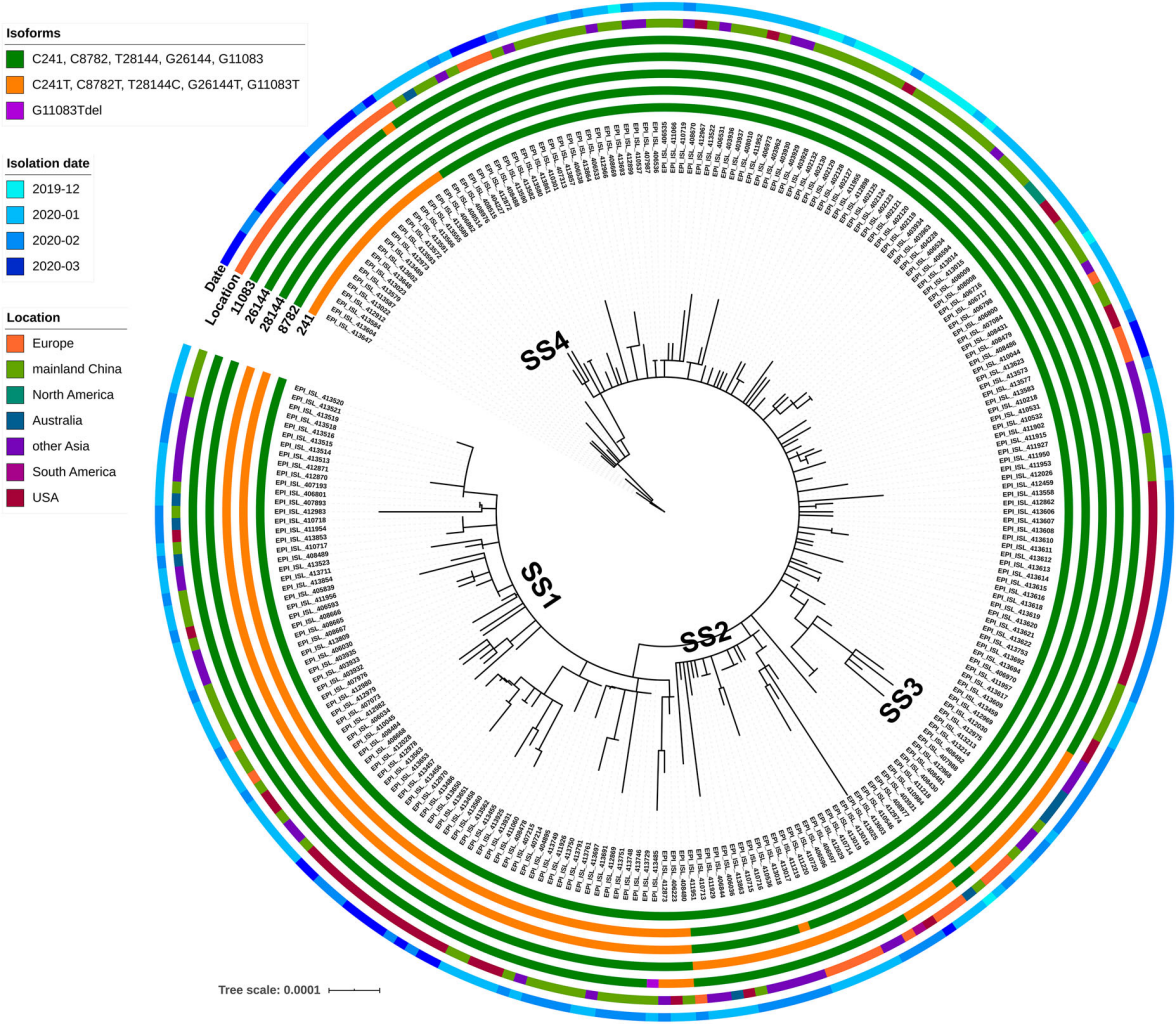
Phylogentic analysis of SARS-CoV-2 genomes. Four super-spreader clusters (SSs) were identified. Each SS was found to exhibit a signature mutation profile.

To trace the evolution process and identify the common ancestor of 247 strains of SARS-CoV-2 collected worldwide, root-to-tip regression scatter plots was conducted among all SARS-CoV-2 genomes, with *R*^2^ being found to be 0.23, suggesting that these 247 viral sequences shared a common recent ancestor (Figure S1a). The date of the most recent common ancestor (tMRCA) of all reported SARS-CoV-2 viruses was 12 November 2019, suggesting that this virus emerged recently (Figure S1a). A total of 379 nucleotide mutations were identified among these 247 sequences based on sequence alignment, among which G^11083^T (*n *= 5), T^3^G (*n *= 3), G^29864^A (*n *= 3), C^29870^A (*n *= 3), A^1^T (*n *= 2), A^4^T (*n *= 2), T^4402^C (*n *= 2), G^5062^T (*n *= 2), T^18603^C (*n *= 2) and G^22661^T (*n *= 2) were the most homoplasic mutations (Figure 1, Table S1). A total of 147 strains were found to contain single amino acid change, with the majority of such changes being located within ORF1ab (*n *= 104). The L^3606^F change was detected in two viral sequences, while other mutations occurred only once. Mutations that result in amino acid changes include single substitution in the S protein (*n *= 19, D^614^G, L^752^F, F^32^I, H^655^Y, V^483^A, F^157^L, V^615^L, K^202^N, S^939^F, F^797^C, A^930^V, R^408^I, V^367^F, Q^409^E, S^254^F, A^435^S, D^1146^E, S^247^R and P^1143^L), ORF3a (*n *= 8, E^191^G, G^76^S, K^61^N, V^259^L, T^176^I, L^140^V, T^269^M and V^88^L), N protein (*n *= 6, K^247^I, S^194^L, P^46^S, S^327^L, E^378^Q and D^343^V), ORF8 (*n *= 4, T^11^I, L^84^S, S^97^N and S^67^F), ORF7a (*n *= 3, P^34^S, Q62* and H^73^Q), ORF10 (*n *= 2, P^10^S and I^13^M) and E protein (*n *= 1, S^6^L) (Figure S1b). Identification of single amino acid substitutions in SARS-CoV-2 isolates consistently showed that these isolates shared a recent common ancestor but entered diverse evolution paths. The estimated substitution rate of SARS-CoV-2 was 8.90e-04 subs/site/year, which was similar to that of other RNA viruses including SARS-CoV, Ebola virus, Zika virus and others, which was found to be at ∼ 1e-3 subs/site/year (http://virological.org/t/phylodynamic-analysis-93-genomes-15-feb-2020/356). Based on this mutation rate, a genome of 29 kb (kilobase) of SARS-CoV-2 will end up with ∼26 mutations per genome per year, suggesting that within the four months’ study period, the number of mutations in each genome should not exceed ten if all test isolates emerged as a result of natural evolution of a single SARS-CoV-2 strain.

### Multiple origins of SARS-CoV-2 in Wuhan, China

To shed light on the evolution trend of SARS-CoV-2, we analysed the time-dependent changes in mutation profiles of the test strains in detail. A total of 16 viral genomes collected before 1 January 2020, were included (Table S2). All of these 16 genomes were obtained from Wuhan, with half being from Huanan Seafood Wholesale Market (HNSM). Six genomes contained identical sequences, four of which belonged to isolates obtained from HNSM. Compared to these six viral genomes, others displayed various mutation profiles which comprised 1 to 6 mutations in the genome. We therefore set these six genomes as reference genome for subsequent analyses. Two earliest viral genomes reported on 24 and 26 December 2019 were found to harbour two and three mutations when compared to the reference viral genome, respectively. Four viral genomes from HNSM also contained two mutations with different profiles, suggesting that the original SARS-CoV-2 strain might have been circulating in HNSM for a certain period of time and underwent mutational changes in different intermediate hosts before infecting human (Table S1). These observations suggested that HNSM was not the only origin of the COVID-19 outbreak, instead the market might only serve as a medium in which transmission of this virus to human first occurred. The original virus was transmitted to various provinces in China subsequently, including Guangdong, Zhejiang, Anhui, Jiangsu and Chongqing, and then to other countries such as Japan, Taiwan, Thailand and USA in the following month (January 2020). The viral genomes reported initially from USA were those of viruses recovered from patients in the Princess Diamond Cruise, confirming that the original virus was the one that caused the outbreak in this cruise; such view is consistent with the finding that identical genomes were reported in Japan, where the cruise ship was docked. A total of 26 out of the 247 genome sequences tested contain one mutation. Unless isolated from the same location, most of these genomes exhibit unique mutational profile. Five sequences from the Princess Diamond cruise ship were found to exhibit unique mutation profiles, thus further suggesting that the virus could undergo adaptive evolution during the transmission process, generating a number of genetic variants. It should be noted that these genome sequences were also reported in Wuhan, other parts of China and various other countries, confirming that the transmission of the original virus to different parts of the world was accompanied by active but random mutational changes during the process (Table S1).

### Phylogenetic analysis of genome sequences of SARS-CoV-2

Phylogenetic analysis of the 247 SARS-CoV-2 genomes was also performed, with results showing that such viral genomes exhibited highly diverse genetic profiles and that random mutations occurred during the evolution process within the first two months. Interestingly, four distinct clusters of genome sequences could be identified among the 247 genomes, with the rest exhibiting more diverse profiles. These results were consistent with the data of maximum-likelihood phylodynamic analysis shown in Figure 1. Comparison of the mutation profile of each cluster enabled us to discover that all viral genomes in the same cluster were derived from one parental viral strain which bears a signature mutation profile, as such profile could be identified in all offsprings of that parental strain (Figure 1). The first cluster contained two mutations, C^8782^T and T^28144^C; the second cluster contained the mutation G^26144^T; the third cluster contained the mutation G^11083^T; the fourth cluster contained three mutations, C^241^T, C^3037^T and A^23403^G. Tracing the changes in mutation profiles of these viral genomes over time allowed us to visualize the transmission and evolution dynamics of SARS-CoV-2. Since viruses of all of these four clusters exhibited very high potential to undergo global transmission, we define viruses in these four clusters as super-spreader cluster 1 (SS1), 2 (SS2), 3(SS3) and 4(SS4) respectively.

### Evolution and transmission of super-spreader cluster 1 (SS1)

SS1 carried the signature mutation profile of C^8782^T and T^28144^C. The C^8782^T change is a silent mutation, whereas T^28144^C is associated with the amino acid substitution L^84^S in the Orf8 protein. The SS1 viruses were presumably transmitted very efficiently, as a total of 85 out of the 247 (34%) genome sequences tested were found to belong to this cluster as of 3 March 2020. The earliest sequences in this cluster was reported in Wuhan, China on 5 January 2020; another seven were subsequently reported in January and February in different parts of China and Australia, suggesting that widespread transmission of this cluster of viruses occurred (Table 1). The viruses in SS1 were mainly transmitted among Asian countries such as China, Vietnam, Japan, South Korea, Taiwan and Singapore, but were also detectable in North America, in particular the states of California and Washington in USA (Table 1). The viruses in SS1 were also found to rapidly mutate along the transmission paths. Three genome sequences that were reported from Australia, Vietnam and USA on 24, 28 February and 3 March 2020 respectively, were found to harbour a total of 11 mutations. An additional nine mutations were acquired by the parental virus within 50 days (from 5 January to 24 February 2020), with a mutation rate of 2.3e-3 subs/site/year (29 kb genome size), which was much higher than the predicted mutation rate of SARS-CoV-2 (4.057 e-4 subs/site/year) and other coronaviruses such as SARS-CoV and MERS virus. Among viral genomes in this cluster, 43 of the 85 genomes exhibited five or more mutations (Table 1).

**Table 1.** Mutation analysis of genome sequences in super-spreader cluster 1.

Accession ID	Location	Collection date	Origin	Number of mutations / % of sequence homology with reference sequence	Nucleotide changes
EPI_ISL_406801	Asia / China / Hubei / Wuhan	2020-01-05	NC	2 / 99%	☆ (C^8782^T, T^28144^C)
EPI_ISL_407893	Oceania / Australia / New South Wales / Sydney	2020-01-24	NC	2 / 99%	☆
EPI_ISL_412979	China / Hubei / Wuhan	2020-01-18	NC	2 / 99%	☆
EPI_ISL_413691	China	2020-01	NC	2 / 99%	☆
EPI_ISL_413729	China	2020-02	NC	2 / 99%	☆
EPI_ISL_413746	China	2020-02	NC	2 / 99%	☆
EPI_ISL_413748	China	2020-02	NC	2 / 99%	☆
EPI_ISL_413750	China	2020-02	NC	2 / 99%	☆
EPI_ISL_403932	China / Guandong / Shenzhen	2020-01-14	NC	3 / 99%	☆ ╣ (C^8782^T, T^28144^C, C^29095^T)
EPI_ISL_403933	China / Guandong / Shenzhen	2020-01-15	NC	3 / 99%	☆ ╣
EPI_ISL_403935	China / Guangdong / Shenzhen	2020-01-15	NC	3 / 99%	☆ ╣
EPI_ISL_406030	China / Guangdong / Shenzhen	2020-01-10	WH	3 / 99%	☆ ╣
EPI_ISL_406593	Asia / China / Guandong / Shenzhen	2020-01-13	NC	3 / 99%	☆ ╣
EPI_ISL_406223	USA / Arizona / Phoenix	2020-01-22	NC	4 / 99%	☆ ╣G^11083^T
EPI_ISL_413809	China	2020-02	NC	4 / 99%	☆ ╣G^18686^T
EPI_ISL_408666	Japan / Tokyo	2020-01-31	NC	4 / 99%	☆ ╣C^2662^T
EPI_ISL_408665	Japan / Tokyo	2020-01-29	NC	5 / 99%	☆ ╣C^2662^T; C^3792^T
EPI_ISL_408667	Japan / Tokyo	2020-01-31	NC	5 / 99%	☆ ╣C^2662^T, G^29705^T
EPI_ISL_405839	China / Guangdong / Shenzhen	2020-01-11	WH	5 / 99%	☆ ╣C^9561^T; T^15607^C
EPI_ISL_411956	North America / USA / Texas	2020-02-11	NC	7 / 99%	☆ ╣T^18603^C; T^18975^A; A^19175^C; C^27925^T
EPI_ISL_404895	USA / Washington / Snohomish County	2020-01-19	NC	3 / 99%	☆ T^28144^C
EPI_ISL_407976	Europe / Belgium / Leuven	2020-02-03	WH	3 / 99%	☆ A^29863^T
EPI_ISL_408480	China / Yunnan / Kunming	2020-01-17	NC	3 / 99%	☆ G^11083^T
EPI_ISL_408489	Taiwan / Taipei	2020-01-31	WH	3 / 99%	☆ G^11528^S
EPI_ISL_410535	Singapore	2020-02-03	NC	3 / 99%	☆ G^28878^A
EPI_ISL_411926	Taiwan / Taipei	2020-01-24	NC	3 / 99%	☆ A^29889^G
EPI_ISL_413854	China / Guangdong	2020-01-30	NC	3 / 99%	☆ C^6501^T
EPI_ISL_411060	China / Fujian	2020-01-21	NC	3 / 99%	☆ ╧ (C^8782^T, T^28144^C, C^18060^T)
EPI_ISL_407214	USA / Washington	2020-01-25	NC	3 / 99%	☆ ╧
EPI_ISL_407215	USA / Washington	2020-01-25	NC	3 / 99%	☆ ╧
EPI_ISL_408478	China / Chongqinq / Yongchuan	2020-01-21	NC	5 / 99%	☆ ╧ C^29200^T, C^1342^T
EPI_ISL_413456	USA / Washington / King County	2020-02-20	NC	5 / 99%	☆ ╧ ╤ (C^8782^T, T^28144^C, C^18060^T, C^17747^T, A^17858^G)
EPI_ISL_413560	USA / Washington	2020-02-28	NC	5 / 99%	☆ ╧ ╤
EPI_ISL_412970	USA / Washington / Snohomish County	2020-02-24	NC	6 / 99%	☆ ╧ ╤C^5784^T
EPI_ISL_413457	USA / Washington	2020-02-29	NC	6 / 99%	☆ ╧ ╤C^20^S
EPI_ISL_413458	USA / Washington	2020-03-01	NC	6 / 99%	☆ ╧ ╤T^20281^C
EPI_ISL_413563	USA / Washington	2020-03-03	NC	6 / 99%	☆ ╧ ╤C^9430^A
EPI_ISL_413650	USA / Washington	2020-03-05	NC	6 / 99%	☆ ╧ ╤ T^23010^C
EPI_ISL_413651	USA / Washington	2020-03-05	NC	6 / 99%	☆ ╧ ╤ T^23010^C
EPI_ISL_413653	USA / Washington	2020-03-05	NC	6 / 99%	☆ ╧ ╤ A^6^T
EPI_ISL_413455	USA / Washington	2020-02-28	NC	8 / 99%	☆ ╧ ╤ T^29867^A, G^29868^A, C^29870^A
EPI_ISL_413486	USA / Washington	2020-03-01	NC	8 / 99%	☆ ╧ ╤ A^3406^C, C^5784^T, C^23525^T,
EPI_ISL_413925	USA / California / San Francisco	2020-03-05	GPCS	8 / 99%	☆ ╧ ╤C^23185^T, A^3046^G, A^16467^G
EPI_ISL_413931	USA / California / San Francisco	2020-03-05	NC	9 / 99%	☆ ╧ ╤ A^3046^G, A^16467^G, G^16975^T, C^23185^T
EPI_ISL_413562	USA / Washington	2020-03-02	NC	10 / 99%	☆ ╧ ╤ C^313^del, C^9180^T, G^29864^A, T^29867^A, G^29868^A
EPI_ISL_413652	USA / Washington	2020-03-05	NC	11 / 99%	☆ ╧ ╤ T^23010^C, G^29861^A, G^29864^C, T^29867^A, G^29868^C, C^29870^A
EPI_ISL_407193	South Korea / Gyeonggi-do	2020-01-25	NC	4 / 99%	☆ ┫(C^8782^T, T^28144^C, T^4402^C; G^5062^T)
EPI_ISL_412870	South Korea/ Seoul	2020-01-30	NC	4 / 99%	☆ ┫
EPI_ISL_413513	South Korea	2020-02-27	NC	4 / 99%	☆ ┫
EPI_ISL_413514	South Korea	2020-02-27	NC	4 / 99%	☆ ┫
EPI_ISL_413515	South Korea	2020-02-27	NC	4 / 99%	☆ ┫
EPI_ISL_413516	South Korea	2020-02-27	NC	4 / 99%	☆ ┫
EPI_ISL_413518	China / Beijing	2020-01-26	NC	4 / 99%	☆ ┫
EPI_ISL_413519	China / Beijing	2020-01-28	NC	4 / 99%	☆ ┫
EPI_ISL_413521	China / Beijing	2020-01-28	NC	4 / 99%	☆ ┫
EPI_ISL_413520	China / Beijing	2020-01-28	NC	5 / 99%	☆ ┫A^29301^T
EPI_ISL_412871	South Korea / Seoul	2020-01-31	NC	6 / 99%	☆ ┫C^1779^T, C^15017^T
EPI_ISL_410718	Queensland / Gold Coast	2020-02-05	NC	4 / 99%	☆╠ (C^8782^T, T^28144^C, G^28878^A; G^29742^A)
EPI_ISL_411954	USA / California	2020-02-06	NC	4 / 99%	☆╠
EPI_ISL_413853	China / Guangdong	2020-01-30	NC	4 / 99%	☆╠
EPI_ISL_410717	Australia / Queensland / Gold Coast	2020-02-05	NC	5 / 99%	☆╠ T^18603^C
EPI_ISL_412978	China / Hubei / Wuhan	2020-01-17	NC	4 / 99%	☆ C^12141^A, C^23816^T
EPI_ISL_413711	China	2020–02	NC	4 / 99%	☆ C^6501^T, C^16887^T
EPI_ISL_413523	India / Kerala	2020-01-31	China	6 / 99%	☆ A^1691^G, C^6501^T, C^16877^T, C^24351^T
EPI_ISL_413749	China	2020-02	NC	4 / 99%	☆ C^14768^T, A^17805^T
EPI_ISL_413858	China / Guangdong	2020-01-30	NC	4 / 99%	☆ A^27749^N, G^27750^N
EPI_ISL_412980	China / Hubei / Wuhan	2020-01-18	NC	5 / 99%	☆ T^18996^C, C^24370^T, T^29029^C
EPI_ISL_407071	Europe / England	2020-01-29	NC	5 / 99%	☆ T^22586^Y; T^23605^G; T^28144^C
EPI_ISL_407073	Europe / England	2020-01-29	NC	5 / 99%	☆ T^23605^G; T^18488^C, A^29596^G
EPI_ISL_412982	China / Hubei / Wuhan	2020-02-07	NC	5 / 99%	☆ G^5657^A, A^23403^G, A^25725^G,
EPI_ISL_413697	China	2020-02	NC	5 / 99%	☆ C^207^T, T^946^C, A^11430^G
EPI_ISL_413751	China	2020-02	NC	5 / 99%	☆ TTT^27792-27794^del
EPI_ISL_413761	China	2020-02	NC	5 / 99%	☆ C^207^T, T^946^C, A^11430^G
EPI_ISL_408484	China / Sichuan / Chengdu	2020-01-15	NC	6 / 99%	☆ ▌T^19190^A; C^24034^T
EPI_ISL_406034	USA / California / Los Angeles	2020-01-23	NC	7 / 99%	☆ ▌G^1548^A; C^24034^T, A^28792^T
EPI_ISL_410045	USA / Illinois	2020-01-28	NC	7 / 99%	☆ ▌T^490^A; C^3177^T, C^24034^T;
EPI_ISL_412028	Hong Kong	2020-01-22	NC	7 / 99%	☆ ▌C^1663^T, G^22661^T, G^29862^T
EPI_ISL_408668	Vietnam / Thanh Hoa	2020-01-24	NC	11 / 99%	☆ ▌A^27^T; C^28^del; C^24034^T; T^29858^C; G^29861^C; G^29864^del; T^29867^A
EPI_ISL_407896	Queensland / Gold Coast	2020-01-30	NC	7 / 99%	☆ A^21949^M; C^24790^T; C^25587^T; G^28878^A; G^29742^A
EPI_ISL_412873	South Korea / Chungcheongnam-do	2020-02-06	NC	7 / 99%	☆ T^3086^C, C^6255^T, G^11083^T, G^17122^A, A^29871^G
EPI_ISL_413791	China	2020-02	NC	7 / 99%	☆ C^207^T, T^946^C, A^11430^G, A^16474^G, C^25000^A
EPI_ISL_404253	USA / Illinois / Chicago	2020-01-21	NC	8 / 99%	T^490^W; C^3177^Y; C^24034^Y; T^26729^Y; G^28077^Y; C^28854^Y
EPI_ISL_412869	South Korea /Seoul	2020-01-30	NC	8 / 99%	☆ A^1740^C, T^8767^C, C^17104^T, G^26167^T, G^29593^A, A^29869^G
EPI_ISL_412983	China / Hubei / Tianmen	2020-02-08	NC	9 / 99%	☆ A^3175^G, G^3179^A, C^14422^T, C^14585^T, G^23405^C, C^28315^T, T^29680^K
EPI_ISL_413485	China / Anhui / Suzhou	2020-01-24	NC	9 / 99%	☆ A^4^T, C^2189^T, T^3086^C, A^5094^G, G^11083^del, C^16049^T, G^17122^A,
EPI_ISL_407894	Queensland / Gold Coast	2020-01-28	NC	11 / 99%	☆ A^6604^R; C^13681^M; A^13682^M; C^13684^M; T^13686^K; G^13687^K; A^13693^W; G^28878^A; G^29742^A

☆, C^8782^T, T^28144^C; ╣, C^29095^T; ╧ C^18060^T; ╤ C^17747^T, A^17858^G; ┫, T^4402^C; G^5062^T; ╠, G^28878^A; G^29742^A; ▌, T^26729^C;

NC, Not confirmed;

Detailed analysis of mutation profiles of the genome sequences in SS1 enables us to trace the evolution routes of these viruses in specific region. In Washington State, USA, a genome sequence with three mutations, C^18060^T, C^8782^T and T^28144^C, was reported on 25 January 2020. A virus carrying these three mutations was reported in Fujian, China on 21 January 2020, suggesting that the virus might have originated from Fujian, China (Table 1). This virus was then further transmitted in the Washington area and continued to acquire mutations. Twelve genome sequences reported between 1 and 5 March 2020 in the states of Washington and California contained 6–11 mutations. These data represent direct evidence of active evolution that results in a large number of mutational changes during the process of transmission of a single virus within a short period of time. In addition, a genome sequence which has two additional mutations when compared to the original virus in this cluster, but were different from those in genome sequences in Washington, was reported in Sichuan, China, suggesting that the same parental virus was also transmitted across China during this period (Table 1).

### Evolution and transmission of super-spreader 2 (SS2)

SS2 carried the signature mutation G^26144^T, which resulted in the G^251^V amino acid substitution in Orf 3 protein of SARS-CoV-2. The first viral genome in this cluster was reported in Australia on 25 January 2020. Among the 247 sequences tested, a total of 28 (11.2%) were found to belong to SS2. The parental virus had acquired different mutations and had been disseminated to various Asian countries, North America (USA), Europe, South America (Brazil) and Australia. Viruses in this cluster seemed to be extensively transmitted by the end of January and lasted till early February. By the end of February, however, transmission efficiency of such viruses seemed to have dropped, as only 4 of 28 sequences reported during the period 26 February to 3 March 2020 belong to this cluster. Viruses in this cluster were also found to have significantly mutated with a total of 12 mutations observed in one strain isolated in Washington on 27 February 2020 (Table 2). Our data showed that as many as 11 additional mutations were acquired by the parental virus within a 30 days period (from 25 January to 27 February 2020), representing a mutation rate of 4.6e-3 subs/site/year (29 kb genome size), which was much higher than the predicted mutation rate of SARS-CoV-2 (8.0e-4 subs/site/year).

**Table 2.** Mutation analysis of genome sequences in super-spreader cluster 2.

Accession ID	Location	Collection date	Origin	Number of mutations / % of sequence homology with reference sequence	Nucleotide changes
EPI_ISL_408977	Australia / Sydney	2020-01-25	NC	1 / 99%	• (G^26144^T)
EPI_ISL_406036	USA / California	2020-01-22	NC	2 / 99%	• C^17000^T
EPI_ISL_412029	Hong Kong	2020-01-30	NC	2 / 99%	• T^13929^C
EPI_ISL_413863	China / Guangdong	2020-02-01	NC	2 / 99%	• C^22787^G
EPI_ISL_406596	France / Paris	2020-01-23	NC	2 / 99%	• ▽ (G^26144^T, G^22661^T)
EPI_ISL_406597	France / Paris	2020-01-23	WH	2 / 99%	• ▽
EPI_ISL_410720	France / Paris	2020-01-23	NC	2 / 99%	• ▽
EPI_ISL_411219	France / Paris	2020-01-28	NC	2 / 99%	• ▽
EPI_ISL_411220	France / Paris	2020-01-28	NC	2 / 99%	• ▽
EPI_ISL_410713	Singapore	2020-01-27	NC	2 / 99%	• C^28849^T
EPI_ISL_410714	Singapore	2020-02-03	NC	2 / 99%	• C^21859^T
EPI_ISL_410536	Singapore	2020-02-06	NC	2 / 99%	• ▾ (G^26144^T, C^21859^T)
EPI_ISL_410715	Singapore	2020-02-04	NC	2 / 99%	• ▾
EPI_ISL_410716	Singapore	2020-02-04	NC	2 / 99%	• ▾
EPI_ISL_410546	Italy / Rome	2020-01-31	HB	2 / 99%	• ◇ (G^26144^T, G^11083^T)
EPI_ISL_412974	Italy / Rome	2020-01-29	NC	2 / 99%	• ◇
EPI_ISL_410545	Italy / Rome	2020-01-29	HB	3 / 99%	• A^2269^T; G^11083^N;
EPI_ISL_413603	Finland / Helsinki	2020-03-03	NC	4 / 99%	• ◇ C^14805^T, G^29405^C
EPI_ISL_413016	Brazil / Sao Paulo	2020-02-28	Italy	5 / 99%	• ◇ C^2388^T, C^14805^T, T^17247^C,
EPI_ISL_413019	Switzerland / Zurich	2020-02-26	NC	9 / 99%	• ◇, G^11084^TTTin, C^14805^T, T^17247^C, C^24378^T, C^26894^T
EPI_ISL_413025	USA / Washington	2020-02-27	NC	12 / 99%	• ◇, A^35^T, C^36^T, T^2446^C, C^3411^T, G^5572^T, C^14805^T, G^29864^A, T^29867^A, G^29868^A, C^29870^A
EPI_ISL_406031	Taiwan / Kaohsiung	2020-01-23	NC	4 / 99%	• G^16188^T; A^25964^G; ^29877^Tin
EPI_ISL_413018	South Korea	2020-02-06	NC	4 / 99%	• A^2707^G, G^26640^T, T^26677^C
EPI_ISL_412116	England	2020-02-09	NC	5 / 99%	• A^2470^G, C^2558^T, G^11083^N, C^14805^T,
EPI_ISL_413017	South Korea	2020-02-06	NC	6 / 99%	• T^4402^C, G^5062^T, G^26640^T, T^26677^C, T^28144^C
EPI_ISL_411951	Sweden	2020-02-07	NC	7 / 99%	• G^2717^A; A^9274^G; C^13225^G; T^13226^C; A^17376^G; T^23952^G;
EPI_ISL_411929	South Korea	2020-01	WH	9 / 99%	• G^2971^T; C^6031^T; C^12115^T; T^15597^C; C^20936^T; C^22224^G; G^25775^T; T^26354^A
EPI_ISL_406844	Australia / Victoria	2020-01-25	NC	13 / 99%	• T^19065^C; T^22303^G; ^29750-29759^del

○, G^26144^T; ▽, G^22661^T; ▾, C^21859^T; ◇, G^11083^T;

### Evolution and transmission of super-spreader 3 (SS3)

SS3 carried the signature mutation G^11083^T, which caused the L^3606^F amino acid substitution in the Orf 1 protein of SARS-CoV-2. The first viral genome in this cluster was reported on 18 January 2020 in Chongqing, China. A total of 22 such genome sequences were reported so far, accounting for 9% of the 247 SARS-CoV-2 sequences documented in the GISAID database as of 5 March 2020. It has since been transmitted to several Asian countries including Singapore and Japan, as well as Europe, USA and Australia (Table 3). Like SS1 and SS2, viruses in this cluster were also found to mutate efficiently, with one genome reported on Febuary 27, 2020 from Washington, USA, carrying 12 mutations. Our data showed that a total of eleven mutations were acquired by the parental virus within a 40 days’ period (from 18 January to 27 February 2020), with a mutation rate of 2.8e-3 subs/site/year (29 kb genome size), which was again much higher than the predicted mutation rate of SARS-CoV-2 (8.0e-4 subs/site/year). Curiously, there is no virus of this cluster being reported in Iran, a country with one of the highest incidence of SARS-CoV-2 infections. However, two genome sequences from Australia, which belong to viruses recovered from patients with travel history to Iran, were reported, suggesting that this cluster of virus might also contribute to the outbreaks in Iran. In addition, the first genome sequence from Brazil, which might have originated from Italy, also belonged to this cluster (Table 3).

**Table 3.** Mutation analysis of genome sequences in super-spreader cluster 3.

Accession ID	Location	Origin	Collection date	Number of mutations / % of sequence homology with reference sequence	Nucleotide changes
EPI_ISL_408481	China / Chongqing	NC	2020-01-18	1 / 99%	☉ (G^11083^T)
EPI_ISL_407988	Singapore	NC	2020-02-01	1 / 99%	☉
EPI_ISL_412968	Japan	NC	2020-02-10	1 / 99%	☉
EPI_ISL_410546	Italy / Rome	HB	2020-01-31	2 / 99%	☉ G^26144^T
EPI_ISL_412030	Hong Kong	NC	2020-02-01	2 / 99%	☉ G^29841^A
EPI_ISL_412969	Japan	NC	2020-02-10	2 / 99%	☉ C^29635^T
EPI_ISL_412974	Italy / Rome	NC	2020-01-29	2 / 99%	☉ G^26144^T
EPI_ISL_408430	France / Paris	NC	2020-01-29	3 / 99%	☉ ◎ (G^11083^T, C^1190^T, C^9438^T)
EPI_ISL_410984	France / Paris	NC	2020-01-29	3 / 99%	☉ ◎
EPI_ISL_411218	France / Paris	NC	2020-02-02	3 / 99%	☉ ◎
EPI_ISL_408480	China / Yunnan / Kunming	NC	2020-01-17	3 / 99%	☉ ♁ (G^11083^T, C^8782^T, T^28144^C)
EPI_ISL_406223	USA / Arizona / Phoenix	NC	2020-01-22	4 / 99%	☉ ♁ C^29095^T
EPI_ISL_412873	South Korea	NC	2020-02-06	7 / 99%	☉ ♁ T^3086^C, C^6255^T, G^17122^A, A^29871^G
EPI_ISL_413485	China / Anhui / Suzhou	NC	2020-01-24	9 / 99%	☉ ♁ A^4^T, C^2189^T, T^3086^C, A^5094^G, C^16049^T, G^17122^A
EPI_ISL_413603	Finland / Helsinki	NC	2020-03-03	4 / 99%	☉ $ G^26144^T, G^29405^C
EPI_ISL_413016	Brazil / Sao Paulo	Italy	2020-02-28	5 / 99%	☉ $ C^2388^T, T^17247^C, G^26144^T
EPI_ISL_413025	USA / Washington	NC	2020-02-27	12 / 99%	☉ $ A^35^T, C^36^T, T^2446^C, C^3411^T, G^5572^T, G^26144^T, G^29864^A, T^29867^A, G^29868^A, C^29870^A
EPI_ISL_413214	Australia Sydney	NC	2020-02-29	5 / 99%	☉ % G^29374^A
EPI_ISL_412975	Australia Sydney	Iran	2020-02-28	6 / 99%	☉ % G^4255^A, A^20047^G
EPI_ISL_413213	Australia / Sydney	Iran	2020-02-29	7 / 99%	☉ % C^884^T, G^8653^T, C^24704^T
EPI_ISL_408482	Shandong / Qingdao	NC	2020-01-19	7 / 99%	☉ % C^7299^T; C^27612^G; T^28688^C
EPI_ISL_413589	Netherlands / Utrecht	NC	2020-03-01	8 / 99%	☉ C^241^T, G^2527^T, C^3037^T, C^6428^T, C^14408^T, A^23403^G, A^25575^C

☉, G^11083^T; ◎, C^1190^T, C^9438^T; ♁, C^8782^T, T^28144^C; $, C^14805^T; %, G^1397^A, T^28688^C, G^29742^T.

### Evolution and transmission of super-spreader 4 (SS4)

SS4 carried a signature mutation profile that consists of three mutations: C^241^T, C^3037^T and A^23403^G. The C^241^T and C^3037^T changes are silent mutations, whereas A^23403^G results in the D^614^G substitution in the spike (S) protein of SARS-CoV-2. SS4 viruses were found to be transmitted only in Europe, with the exception of one genome reported from Mexico, in which the patient had travel history from Italy. Viruses in SS4 were responsible for the explosive increase in incidence of COVID-19 in Europe in March (Table 4). Compared to SS1, 2 and 3, viruses in SS4 were reported more recently, mostly from the end of February to early March. A total of 21 of the 247 genomes examined (8.4%) were found to belong to SS4. Among genome sequences of the four clusters of super-spreaders, none was found to contain only one of the three SS4 mutations or a combination of two of the three mutations, suggesting that the parental viral genome of SS4 could not be identified. The first virus of this cluster, reported on 28 January 2020 in Germany, has acquired another silent mutation, C^14408^T, and was further disseminated to other countries in Europe. This virus was also found to mutate efficiently with seven additional mutations were acquired by the parental virus within 30 days (from 28 January to 27 February 2020), with a mutation rate of 2.8e-3 subs/site/year (29 kb genome size), which was much higher than the predicted mutation rate of SARS-CoV-2 (8.0e-4 subs/site/year). At a later stage, SS4 viruses became the most efficient in causing transmission in Europe. Among a total of 28 genomes reported between 20 February to 3 March 2020, 20 (71%) belonged to this spreader (Table 4).

**Table 4.** Mutation analysis of genome sequences in super-spreader cluster 4.

Accession ID	Location	Origin	Collection date	Number of mutations / % of sequence homology with reference sequence	Nucleotide changes
EPI_ISL_406862	Germany / Bavaria / Munich	NC	2020-01-28	3 / 99%	△ (C^241^T, C^3037^T, A^23403^G)
EPI_ISL_413555	United Kingdom / Wales	NC	2020-02-27	4 / 99%	△ # (C^241^T, C^3037^T, A^23403^G, C^14408^T)
EPI_ISL_413566	Netherlands / Blaricum	NC	2020-03-02	4 / 99%	△ #
EPI_ISL_413591	Netherlands / Zeewolde	NC	2020-03-02	4 / 99%	△ #
EPI_ISL_413593	Luxembourg	NC	2020-02-29	5 / 99%	△ # C^23575^T
EPI_ISL_413648	Portugal	Spain	2020-03-01	5 / 99%	△ # C^29144^T
EPI_ISL_412973	Italy	NC	2020-02-20	6 / 99%	△ # T^29867^N, G^29868^N
EPI_ISL_413489	Italy / Milan	NC	2020-03-03	8 / 99%	△ # A^187^G, A^6956^C, T^29867^N, G^29868^N
EPI_ISL_413602	Finland / Helsinki	NC	2020-03-03	6 / 99%	△ # G^22865^T, C^29585^T
EPI_ISL_413572	Netherlands / Haarlem	NC	2020-03-01	7 / 99%	△ # T^1666^C, C^3037^T, G^25563^T
EPI_ISL_413589	Netherlands / Utrecht	NC	2020-03-01	8 / 99%	△ # G^2527^T, C^6428^T, G^11083^T, A^25575^C
EPI_ISL_413022	Switzerland / Zurich	NC	2020-02-29	7 / 99%	△ # & (C^241^T, C^3037^T, A^23403^G, C^14408^T, G^28881^A, G^28882^A, G^28883^C)
EPI_ISL_413579	Netherlands / Nootdorp	NC	2020-03-03	7 / 99%	△ # &
EPI_ISL_413587	Netherlands / Tilburg	NC	2020-03-03	7 / 99%	△ # &
EPI_ISL_412912	Germany/Baden-Wuerttemberg	Italy	2020-02-25	8 / 99%	△ # & G^10265^A
EPI_ISL_413584	Netherlands / Rotterdam	NC	2020-03-03	8 / 99%	△ # & C^27046^T
EPI_ISL_413647	Portugal	Germany	2020-03-01	8 / 99%	△ # & C^27046^T
EPI_ISL_413604	Finland / Helsinki	NC	2020-03-03	9 / 99%	△ # & C^27046^T, T^29807^C
EPI_ISL_413023	Switzerland / Zurich	NC	2020-02-29	9 / 99%	△ # & A^22168^C,
EPI_ISL_412972	Mexico / Mexico City	Italy	2020-02-27	10 / 99%	△ # & C^13206^G, A^15807^del, G^24268^del,

△, C^241^T, C^3037^T, A^23403^G; #, C^14408^T; &, C^14408^T, G^28881^A, G^28882^A, G^28883^C.

### Temporal and spatial distribution of super-spreaders of SARS-CoV-2

**Figure 2.**
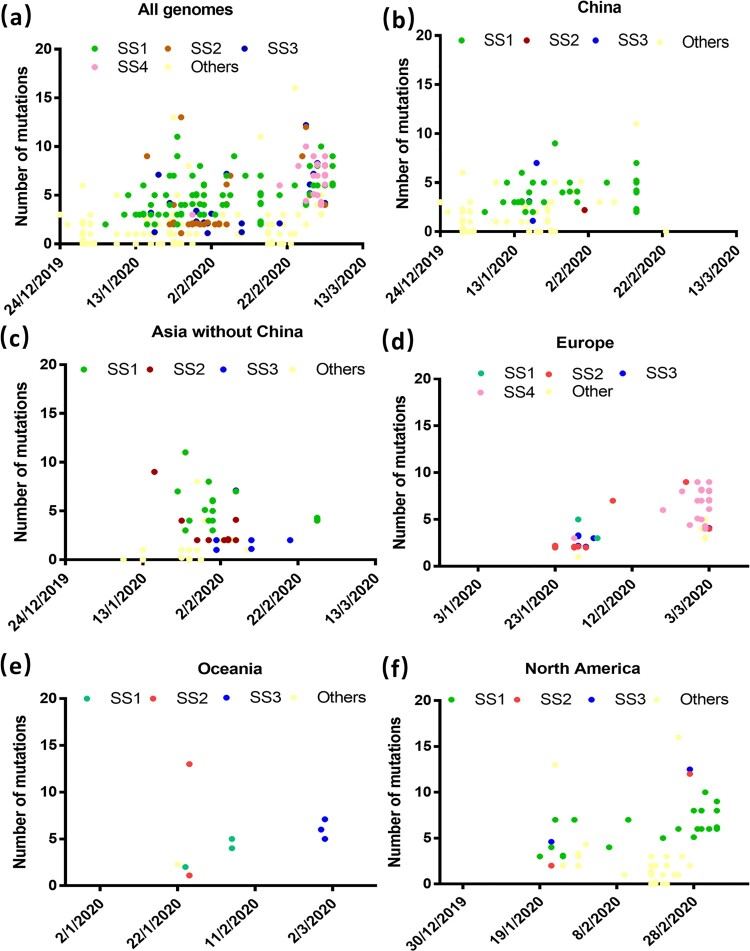
Changes in the distribution pattern and mutation rate of different super-spreader clusters in various continents over time. Distribution of different SSs and their mutations (a) Overall, (b) in China, (c), in Asian countries excluding China, (d) in Europe, (e) in Oceania, and (f) in North America. Two genomes with over 20 mutations were not included to facilitate easy visualization of the graphs.

To better understand the temporal and spatial distribution of these super-transmitters, we plot variation in the types of genome sequences recovered from different continents against time. The original viruses were found to be spreading in the week before the emergence of these super-spreaders. SS1 was the first batch of viruses that emerged and dissemination continued throughout the study period. Other SSs emerged at different time points and transmission also peaked at different dates. Transmission of SS2 and SS3 mainly occurred between mid January to mid February. Transmission of SS4 viruses mainly began at the end of February. Viruses of the four clusters exhibited much higher mutation rate than those which exhibited diverse genetic profiles and could not be allocated into specific genetic cluster when compared to the original genome (Figure 2(a)). SS1 viruses were those which were disseminated extensively in China, in particular in the later stage of the outbreak (Figure 2(b)). SS1, SS2 and SS3 were prevalent in other Asian countries (Figure 2(c)). All the four clusters were involved in the outbreaks in Europe at the early stage, but SS4 was the cluster that eventually transformed the outbreaks in Europe into the pandemic level (Figure 2(d)). In Oceania, SS1 was involved mainly in the early stage of the outbreak, yet SS2 became dominant at a later stage (Figure 2(e)). SS1 and other types of viruses were the major transmitters in the US. SS1 was shown to be transmitted mainly in the states of Washington and California, whereas the other types were mainly transmitted in other states (Figure 2(f), Table S1).

### Distribution of different super-spreader types of most recent SARS-CoV-2 in different parts of the world

Upon completion of analysis of SARS-CoV-2 sequences available in the GISAID database as of 5 March 2020 and identification of the four “super-spreader” type strains, we investigated if viral strains of the four super-spreaders were responsible for the vast majority of subsequent infections. A total of 1539 genome sequences reported after 29 February 2020 were included for a quick analysis to identify the type of these most recent genomes. As shown in Table 6, most of the genomes were reported from USA (968 / 63%) and Europe (441 / 29%), where the pandemic was the most severe. Genomes of the four super-spreaders became detectable in Africa (20 / 1%) and South America (23 / 2%). Among these 1539 genomes, 89% belonged to SS1-4, with SS4 being the most dominant (56%), whereas viruses derived from the original clone that do not belong to any of the four super-spreaders accounted for only 11% of the genomes, and were mainly reported in UK and Netherland. In Africa, SS4 (18/20, 90%) was the major type, with some cases involving patients who had travel history to Europe. In Asia, the major types became SS3 (17/33, 52%) and SS4 (16/33, 48%). In Europe, all the four types were present, with SS4 remaining the dominant one (668/968, 69%). Likewise, all the four super-spreader types were reported in the US, with SS1 (282/441, 62%) and SS4 (137/441, 14%) being more prevalent. In Canada, all types except for SS1 were present. In Oceania, all four SSs were present, with SS3 being more common. In South America, SS1 and SS4 were the most dominant types (Table 5).

**Table 5.** Distribution of different types of SARS-CoV-2 worldwide after 29 February 2020.

Types of viruses	Numbers of genomes (%)	Major areas	Locations / numbers of genomes (%)						
			Africa	Asia	Europe	NA (USA)	NA (Canada)	Oceania	South America
Total	1539		20(1)	33(2)	968(63)	441(29)	11(1)	27(2)	23(2)
Original derivatives	173 (11)	Europe (UK and Netherland)	0(0)	0(0)	158(16)	11(3)	2(2)	0(0)	2(9)
SS1	340 (21)	USA, Europe, South America	1(5)	1(3)	30(3)	282(64)	0(0)	5(19)	8(35)
SS2	172 (11)	Europe	1(5)	0(0)	111(12)	9(2)	2(2)	3(11)	0(0)
SS3	132 (9)	Asia, Europe	0(0)	17(52)	119(12)	12(3)	5(5)	15(56)	0(0)
SS4	856 (56)	Africa, Asia, Europe, Oceania	18(90)	16(48)	663(69)	137(14)	4(4)	5(19)	12(52)

NA, North America.

## Discussion

**Figure 3.**
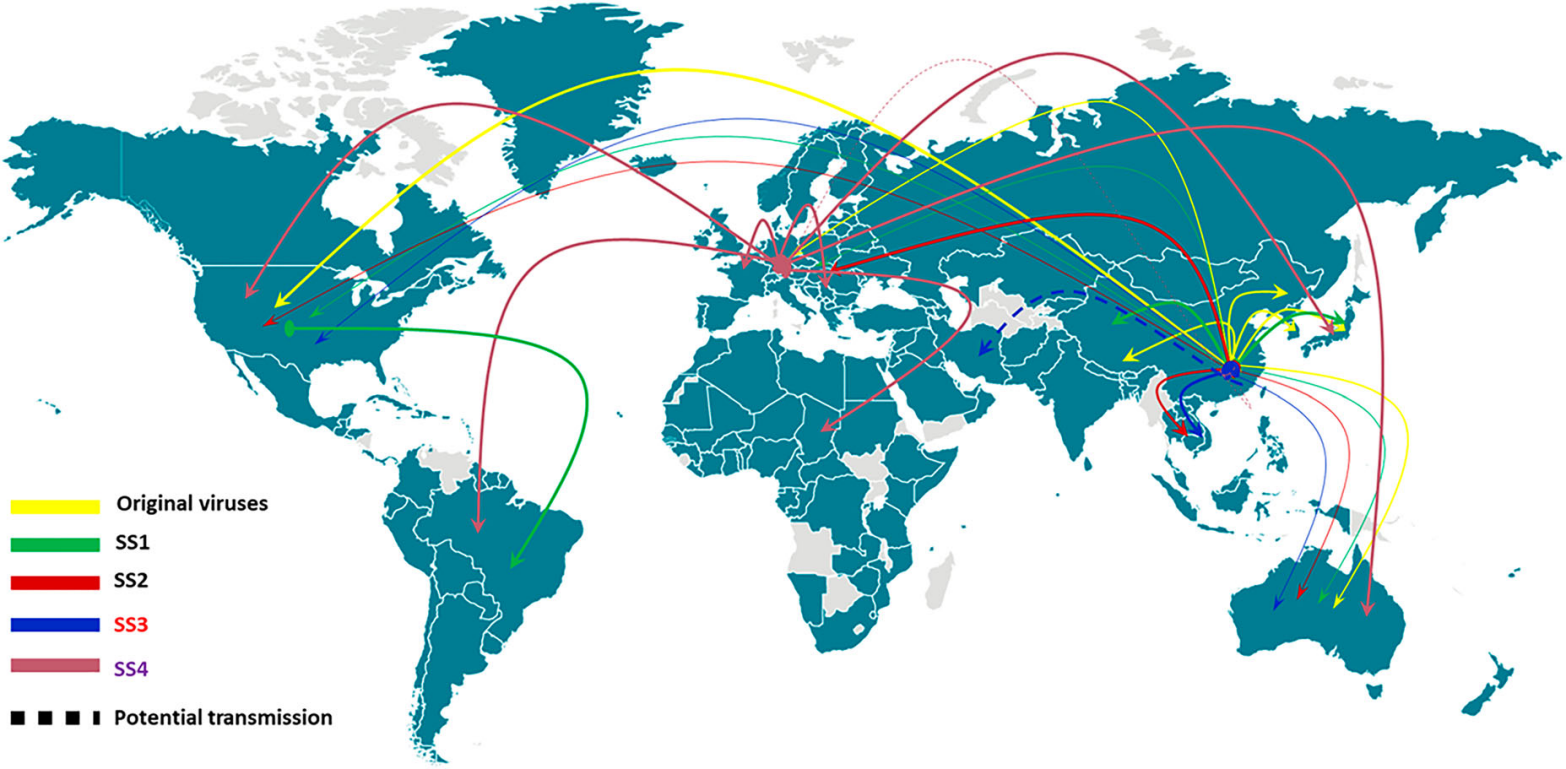
Transmission of super-spreaders and other derivatives of the original SARS-CoV-2 in different parts of world. Derivative strains of the original virus have been transmitted worldwide and contributed to the early outbreak of COVID-19. SS1 strains were transmitted mainly in Asia and the US but were less prevalent in other parts of the world. SS2 and SS3 strains were transmitted mainly in Asian countries other than China, as well as Europe from mid January to mid February. SS4 strains were transmitted mainly in Europe at the beginning of the pandemic and were then transmitted to all over the world.

We conducted detailed and comprehensive analyses of 247 high quality SARS-CoV-2 sequences deposited in the GISAID database during the period December 2019 to 5 March 2020 to provide insight into the evolution and transmission of this novel virus (Figure 3). Our data indicated that the ancestor strain of SARS-CoV-2 could have emerged at a date as early as November, 2019. According to the time line of outbreaks, the original virus from Wuhan city and HNSM was responsible for the initial transmission of SARS-CoV-2 in various countries in January. The origin of the outbreak was not limited to HNSM, instead, those which occurred in multiple sites in Wuhan city might have contributed more significantly to the early transmission events and subsequent dissemination to different parts of China and various countries around the world. These data implied that wild animals sold in HNSM may not be the intermediate host of SARS-CoV-2 as sources other than HNSM are also considered the origin of this virus. Given the fact that multiple patients in Wuhan were simultaneously infected by viruses of different genetic composition in the initial outbreak, we hypothesize that a common wild animal would be the most likely intermediate host. Alternatively, a common environmental factor, such as a faulty sewage system, may be involved. It is necessary to investigate the possible role of a common animal vector or dissemination route in eliciting the initial outbreak that involved multiple SARS-CoV-2 strains.

Interestingly, as the original virus continued to transmit in China and all over the world, it has evolved into four major genetic clusters, namely super-spreader clusters, along with other non-cluster variants derived from the original virus. Each SS cluster carried one or more unique signature mutation(s) which enable us to trace the origin and transmission paths of most subsequently recovered SARS-CoV-2 strains. In the early transmission stage (December 2019 and early January 2020), variants from the original virus were dominant, yet by the end of February and early March, members of four super-spreaders became dominant, with different SSs being prevalent in different regions of the world. SS1 was prevalent in China and other parts of Asia and became the major virus that caused severe outbreaks in Washington and California states in the US and South Korea; SS2 and SS3 were extensively transmitted in other parts of Asia and Europe during the end of January and early February but their prevalence dropped at the end of February and early March, and was replaced by SS4 which also contributed to the pandemic in Europe. Interestingly, SS4 was not reported in China or other parts of the world. The first genome of this cluster was reported in Germany and contributed to the rapid dissemination of SARS-CoV-2 in Europe. Mutation profile with SS4 is unique, with three mutations being observed in the first viral genome. Importantly, the mutation A^23403^G, which results in the D^614^G substitution in the S protein of SARS-CoV-2, was also identified as characteristic genetic change in a highly transmissible strain by Korber et al [20]. Genomes with only one or two of these three mutations were not reported elsewhere. However, these data do not simply imply that SS4 originated from Europe. One limitation of the study is that we can only make assessment using currently available genome sequences. The lack of genome sequence of SS4 in other continents does not necessarily mean that SS4 viruses are not present in other continents. In fact, viral strains carrying the D^614^G substitution have already been recovered in Canada and the USA in March 2020 in our second phase analysis to verify the transmission potential of strains of the four super-spreaders [20]. A second limitation of this study is the lack of data to explain the mechanisms underlying the evolution of various genetic clusters into super-spreaders. Since every super-spreader cluster carries at least one amino acid substitution, whether such amino acid changes enabled SARS-CoV-2 to exhibit superior transmission potential needs to be investigated in future research studies.

Our data also unveiled the genetic features and transmission paths of major viral strains responsible for the current global pandemic of SARS-CoV-2 in detail. For example, in Italy, SS2, SS3 were prevalent in the end of January but gave way to SS4 in February and early March. Similar trends were observable in other countries, with exception of a consistently high proportion of the original viral genomes in Netherland throughout the course of the pandemic. In the US, the original viruses were reported in various states, whereas SS1 was dominant in Washington and California States. Other SS genomes were also sporadically reported in the US. Although data from Iran is not available, two genomes reported from Australia with travel history from Iran were shown to belong to SS3, suggesting that this cluster was responsible for the pandemic in Iran. In Australia, all genomes were reported except SS4.

Our data are consistent with those obtained in a recent study. Analysis of 160 complete genomes of SARS-CoV-2 by Forster et al. identified three central variants, namely Type A, B and C [21]. Type A was the ancestral virus. Type B strains carried the C^8782^T and T^28144^G mutations, which was equivalent to our SS1. Type C, which carried the G^26144^T change, was equivalent to SS2. Forster *et al* found that Type A and C were mainly transmitted in Europe and America, whereas Type B was the most common type in East Asia. Our results are therefore highly consistent with theirs but our works could elucidate the transmission patterns in details. For example, we found that viruses in SS1, or type B according to the work of Forster et al, were mainly transmitted among Asian countries such as China, Japan, South Korea, Taiwan and Singapore, but were also common in North America, in particular the states of California and Washington in USA. Furthermore, we also identified SS3 and SS4, as more viral sequences were included in our analysis. Accuracy of this kind of phylodynamic analysis depends on comprehensiveness of viral genome sequences procured at different stages of infection. Data will inevitably be biased in if genomic sequences of viruses that caused infections in specific countries are under-represented. Nevertheless, we were able to validate the accuracy of our phylodynamic analysis by using the signature mutations as markers for different SSs to determine the relative prevalence of each SS type in 1539 genomes reported in March. The data further confirmed that four SSs continued to be dominant, with around 90% of the genomes belonging to these four SSs, among which SS4 remained the major cluster being disseminated in Europe. This second dataset showed that viruses of SS4 have since been transmitted to other parts of world including Africa, Asia, North America and Oceania. SS1 continued to be the major type in the US but has been transmitted to South America, in particular Brazil. These data appear to suggest that SS1 and SS4 have out-competed SS2 and SS3 and became super-spreader strains responsible for future transmission of SARS-CoV-2.

In conclusion, this study show that four major genetic clusters of viruses have evolved from the original SARS-CoV-2 and have transmitted extensively, each becoming dominant in different parts of the world, and that viruses without any signature mutation of the four super-spreaders appear to be transmitted much less efficiently. These super-spreaders exhibit not only high transmission efficiency, but also high mutation rate without compromising infectivity, posing enormous challenge to the control of future transmission of SARS-CoV-2.

## Supplementary Material

supplementary_figure_1.docx

supplementary_table_2.xlsx

supplementary_table_1.xlsx
