# Supplementary material for: Genetic cluster analysis of SARS-CoV-2 and the identification of those responsible for the major outbreaks in various countries
Source: Emerg Microbes Infect. 2020 Jun 11;9(1):1287–99. doi: 10.1080/22221751.2020.1773745 (PMC7477621; doi:10.1080/22221751.2020.1773745)
Supplement: supplementary_figure_1.docx [file TEMI_A_1773745_SM7274.docx]

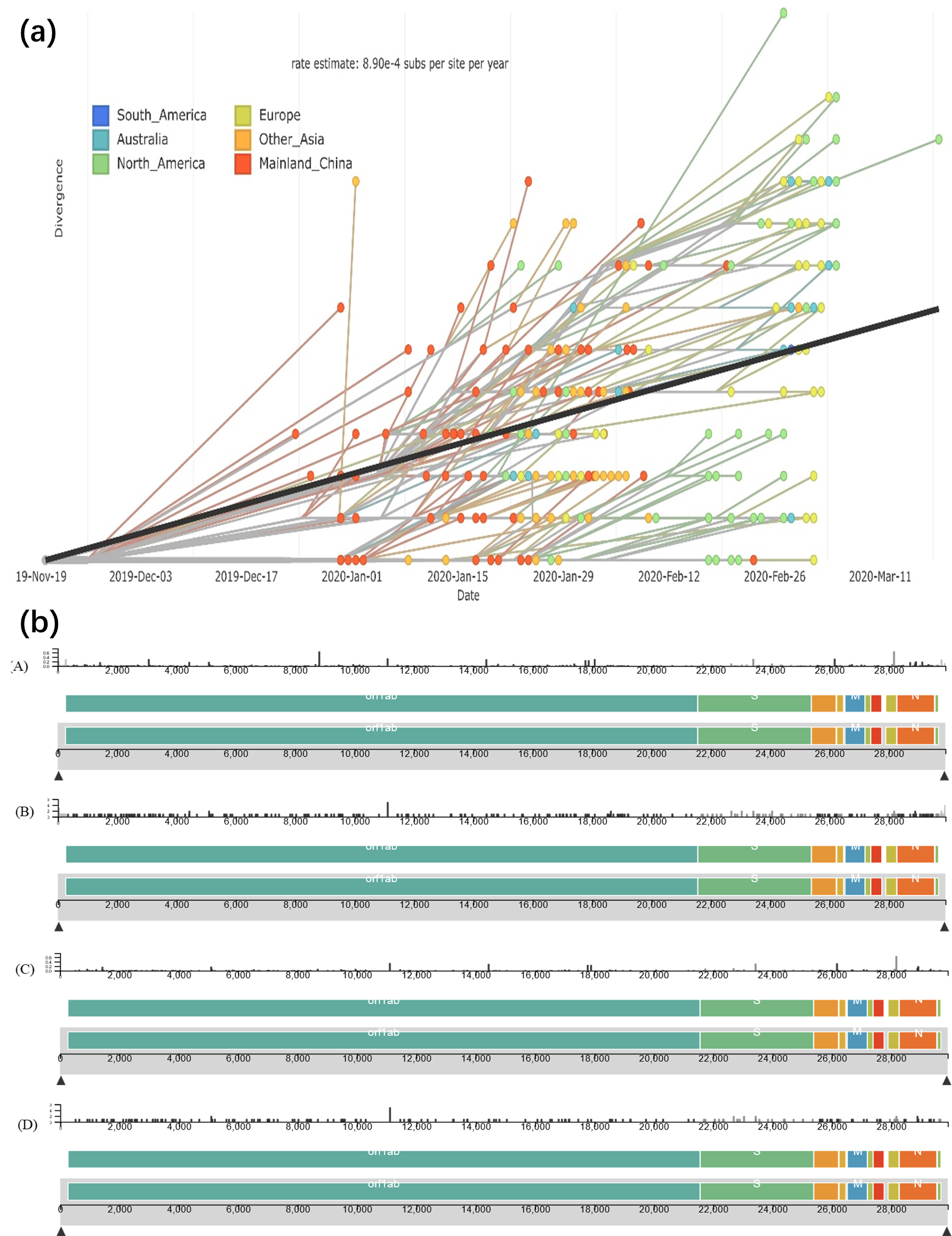


**Supplementary Figure 1. Root-to-tip regression scatter plots and distribution of mutations across the SAR-CoV-2 genomes.** (a) Root-to-tip regression scatter plots of different strains of SARS-CoV-2. (b) Distribution of mutations across the SAR-CoV-2 genome. (A) and (B) illustrated the entropy and events of nucleotide mutations. (C) and (D) illustrated the entropy and events of amino acid mutations. Figure 1. Root-to-tip regression scatter plots of different strains of SARS-CoV-2. Dots in the plot indicate the SARS-CoV-2 isolates used in this study. The color of each dot represents the region of isolation of the corresponding isolate.
